# Texting Teens in Transition: The Use of Text Messages in Clinical Intervention Research

**DOI:** 10.2196/mhealth.3232

**Published:** 2014-11-06

**Authors:** Gwen R Rempel, Ross T Ballantyne, Joyce Magill-Evans, David B Nicholas, Andrew S Mackie

**Affiliations:** ^1^Athabasca UniversityFaculty of Health DisciplinesAthabasca, ABCanada; ^2^University of AlbertaFaculty of NursingEdmonton, ABCanada; ^3^University of AlbertaDepartment of Occupational TherapyEdmonton, ABCanada; ^4^University of CalgaryFaculty of Social WorkCalgary, ABCanada; ^5^University of AlbertaFaculty of Medicine and DentistryEdmonton, ABCanada

**Keywords:** teens, congenital heart disease, text messaging, SMS, qualitative research

## Abstract

**Background:**

The rapidly growing population of young adults living with congenital heart disease (CHD), currently challenging ill-prepared cardiac care systems, presents a novel population in which to consider the use of mHealth. This methodological study was part of a larger study that tested the effectiveness of a clinic-based nursing intervention to prepare teens for transfer from pediatric to adult cardiology care. The intervention included creation of a MyHealth Passport and subsequently SMS (short message service) text messages between the intervention nurse and study participant.

**Objective:**

Our aim was to determine (1) the preference of teens with CHD to be contacted via text message following the nursing intervention, (2) the effectiveness of texting to collect data regarding the use of MyHealth Passport after participation in the intervention, (3) the nature of the texting interaction, and (4) the risks and benefits of texting.

**Methods:**

Participants were recruited through the intervention study (n=24) by either choosing to receive information from the study coordinator through text message, or texting a question to the study nurses. Inclusion criteria were age 15-17 years, diagnosed with moderate or complex heart disease, and currently being followed by the Division of Cardiology at Stollery Children’s Hospital. Exclusion criteria were heart transplantation and/or less than a 6th grade reading and comprehension ability. Text message transcripts were analyzed by qualitative inductive content analysis.

**Results:**

Two-thirds of teens (16/24, 67%) chose text messaging as their preferred contact, making them eligible for the study. Texting was effective in collecting information regarding the MyHealth Passport; all but one teen had their MyHealth Passport on them, and many reported carrying it with them wherever they went. All teens reported showing their MyHealth Passport to at least one person. Seven themes were identified in the texting transcripts: mixing formal and informal language, the passive teen, interaction with health care providers, texting teens in transition, texting as a mechanism to initiate other forms of communication, affirmation, and the nurse as an educator. Benefits of texting were identified as flexibility, ability to respond over time, information presented in byte-sized amounts, and information directly related to patient questions. Risks of texting were identified as the possibility that interactions may not be in-depth, distraction of teen and researcher, and invasiveness.

**Conclusions:**

Text messaging was useful in collecting data regarding the use of the MyHealth Passport. Text messaging resulted in conversations with the teens that were sometimes in-depth and meaningful, especially when combined with other communication modalities. Using text messaging in a manner resulting in full conversations with the patients requires more study and may benefit from protocols and the use of solid theoretical foundations that would standardize the interaction so that more conclusions could be drawn.

## Introduction

Mobile health (mHealth), the delivery of health-related services through mobile communication, is gaining interest in the research community [1]. Texting-based interventions have been evaluated in many areas including violence and neglect [2,3], asthma control [4,5], human immunodeficiency virus/acquired immune deficiency syndrome and sexual health [6-15], exercise and weight control [16-24], diabetes management [25-29], smoking cessation [30-38], oral health [39], increasing attendance at clinics [40-44], communication of medical results [45], screening [46], and mental illness support [47-54]. Few studies deal exclusively with adolescents [8,9,11,20,22,23,26,28,29,31,32,49], and none have examined adolescents living with congenital heart disease (CHD). Additionally, few studies feature two-way discussion between researcher and participant [7,14,15] as the primary method of communication; most studies use an automated text messaging system or send standard messages with varying levels of personalization. There are some studies that include two-way discussions, but these studies do not focus exclusively on this type of interaction.

There is a rapidly growing population of young adults living with CHD; these individuals challenge ill-prepared cardiac care systems [55]. CHD is the most common birth defect and is present in 8-9 of every 1000 newborns [56,57]. Historically, affected individuals died in childhood. Advances in health care currently enable many of these children to survive into adulthood, resulting in increased pressure on the health system to provide care to this emerging population [58]. The situation is further complicated by the fact that over half of these young adults do not attend their follow-up appointments [59] and that many individuals with CHD are ill-informed about their heart conditions and health care [60]. Some young adults hold the misconception that they were cured in childhood [61]. Cardiac care services have begun to address these issues by creating transition programs with the intent of encouraging the movement of adolescents and young adults with chronic medical conditions from pediatric to adult care [62]. Transition, in this instance, is not viewed as a one-time event, but rather a multidimensional process to foster self-management through childhood and adolescence [63].

The objective of Texting Teens in Transition, as part of the larger Congenital Heart Adolescents Participating in Transition Evaluation Research (CHAPTER) Study, was to determine the nature of texting by teens in the context of clinical intervention research. The CHAPTER study is a single site, cluster controlled trial conducted to test whether a nurse-led clinic-based intervention, in addition to usual care, resulted in better “transition readiness” than usual care alone [64]. The CHAPTER study intervention focused on increasing patient knowledge [65-68] and promoting self-management skills [69-71]. During the intervention, a MyHealth Passport was created in addition to activities to increase knowledge and self-efficacy, including review of the teen’s cardiac anatomy, discussion of potential complications, introduction of transition resources (eg, websites), and application of learning through three scenarios and the setting of one education goal.

The MyHealth Passport is a Web-based program in which teens enter information regarding their health care including diagnosis, test results, and physician names [72]. The final document is printed onto a wallet-sized card. The MyHealth Passport fosters knowledge and self-management. By creating the document, the teen learns more about their diagnosis and how to manage their condition. Additionally, the document itself is used by the teen to more effectively interact with health care providers, which is a necessity for self-management. Thus it was important to keep the MyHealth Passport with them. Text messages were used to invite teen commentary regarding their use of the MyHealth Passport.

This paper presents the qualitative analysis of the text messages that determined (1) the preference of teens with CHD to be contacted via text message following the CHAPTER study intervention, (2) the effectiveness of texting to collect data regarding the use of the MyHealth Passport, (3) the nature of the texting interaction, and (4) identified risks and benefits of texting.

## Methods

### Study Design

Participants for this study were recruited through the larger CHAPTER Study. From that study, participants were specifically recruited to this Texting Teens in Transition study in one of two ways. At enrollment in the CHAPTER study, the participants were asked if they wished to receive information from the CHAPTER team through text, email, or phone. Participants were enrolled in Texting Teens in Transition if they indicated a preference to be contacted by text message. Second, at the end of the nurse-led intervention, participants were again invited to indicate their preference between text, email, or phone contact with one of the study nurses following the nurse-led clinical intervention. Participants in the CHAPTER study who indicated a follow-up preference other than text messaging are not included in this paper as it did not relate to the texting interaction. Participants were recruited between January 2011 and May 2012. Inclusion criteria were 15-17 year olds with moderate or complex heart disease [73], who were followed by the Division of Cardiology at the Stollery Children’s Hospital. Teens who had undergone heart transplantation and/or who were below a grade 6 reading and comprehension level were excluded.

### Procedure

At the end of the nurse-led clinical intervention, the nurse welcomed the teen to text back with any questions. The nurse also informed the teen that if the teen had not already contacted the study team in the week following the CHAPTER Study intervention, one of two study nurses (usually the nurse who had interacted with the teen in clinic) would send the participant a text message at the time to which they agreed. If the nurse did not get a response, she sent another text message the following day. If there was still no response, the nurse made no further attempts to contact the teen. The teen was welcome to text back at any time and begin participation.

All interactions began with the nurse addressing any questions the teen had. “Do you have any question?” was the scripted question used to gather data in the group that did not text in with a question. Next, specific questions were asked of the teen regarding the MyHealth Passport. The questions were (1) “Where is your MyHealth Passport now?” and (2) “Have you used your MyHealth Passport or shown it to anyone else?” The interaction was to be predominantly guided by the teen after the nurse gathered the above data. Throughout the study, every effort was made to text the teens outside of school hours.

**Figure 1:**
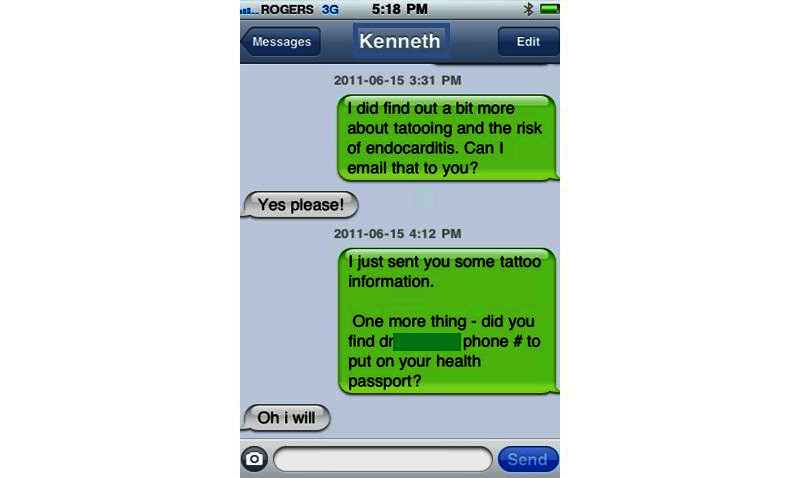
Edit feature.

**Figure 2:**
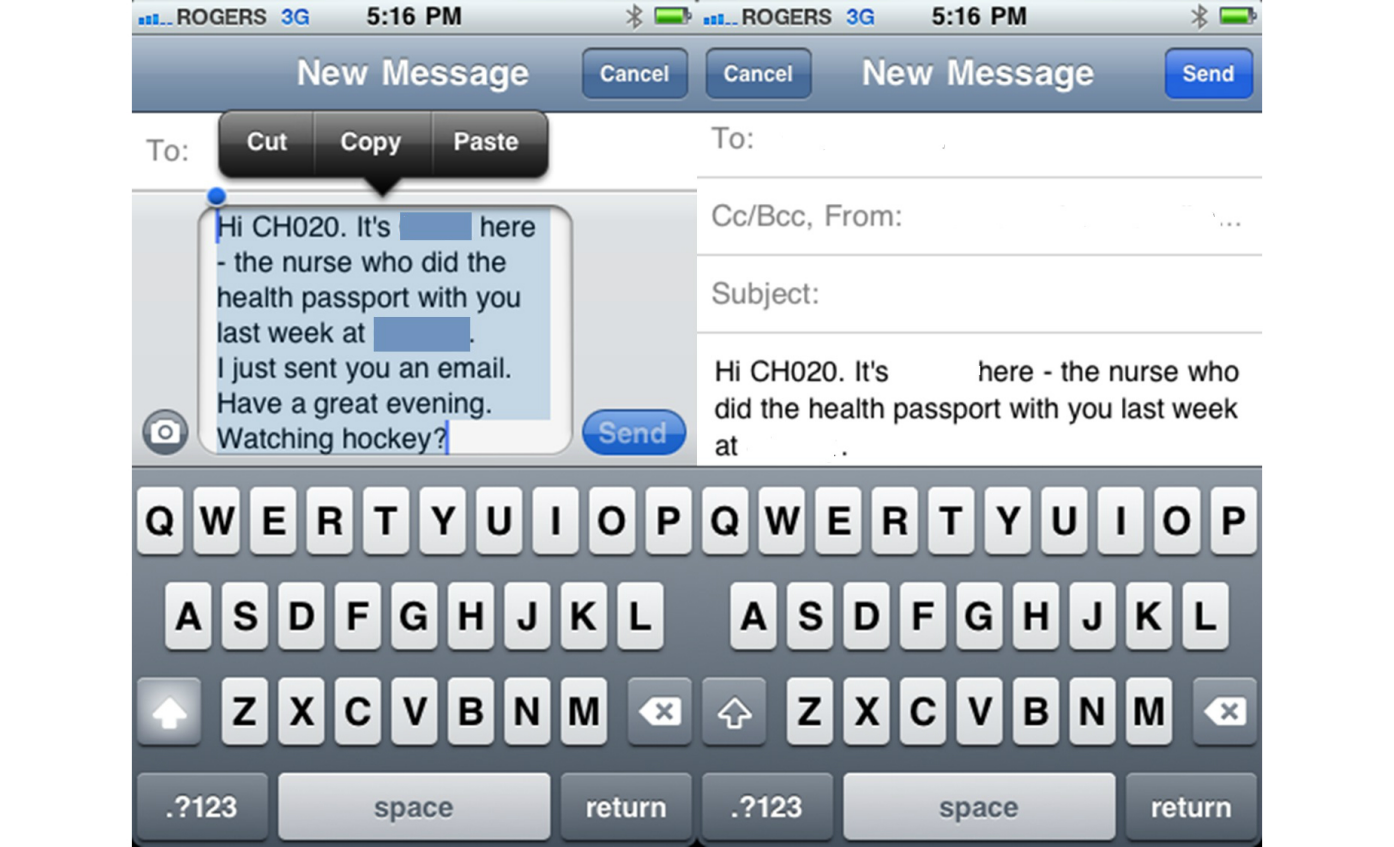
Consolidated texts, anonymized and emailed.

Texting interactions took place on a smartphone used for the study. When the texting interaction between the teen and the nurse was complete, the nurse selected all text messages through the Edit feature (see Figure 1) and forwarded them to the same smartphone. This text message that contained all of the texts was then anonymized before emailing the script of the texting interaction to the research coordinator for data management (see Figure 2). The text messages were then combined into one document and uploaded to a secure server. The messages on the phone were then deleted. The texting transcriptions constituted the study data for analysis as did the field notes (FN) that the nurse recorded after the intervention.

### Data Analysis

The text message transcripts and field notes were analyzed by 2 members of the research team (GR and RB). Code categories and subcategories were identified as per qualitative inductive content analysis [74-76]. The unit of analysis consisted of each texting interaction, defined in this study by at least one reciprocated message between the teen and the nurse. These data were then analyzed for latent content, including temporal separation of messages, formality of language, and sentence formation, as well as manifest content.

### Ethical Considerations

This study was approved through the Health Research Ethics Review Board at the University of Alberta. Both the teen and a parent provided signed informed consent. Identifying information was removed from texts prior to analysis.

## Results

### Overview

Of the teens enrolled in the larger study, two-thirds (16/24, 67%) indicated a preference to be contacted by text message, making them eligible for this substudy. A quarter of teens (6/24, 25%) chose email and 2/24 (8%) chose telephone. Successful contact varied with the highest percentages of contact being established with participants in the texting group (Table 1).

The teens’ preference for texting was also determined by the extent of the texting interactions; they ranged in length from seven messages at the shortest, to 62 messages at the longest. This number included the teen’s and nurse’s texts. The average number of text messages in one interaction was 28. Most interactions were completed on the day contact was initiated. With 3 participants, the interactions occurred over the course of 2 days. All contact with the teens took place between 3:45 pm and 9:00 pm.

**Table 1:** Preference of contact modality indicated by teen and contact made by modality.

Contact modalities	Preference indicated by teen, n (%)	Contact made, n (%)
Text	16/24 (67)	13/16 (81)
Email	6/24 (25)	4/6 (67)
Telephone	2/24 (8)	0/2 (0)

### MyHealth Passport

#### Where is Your Passport Right Now?

All but one of the 24 teens in the study (92%) had their MyHealth Passport on their person; these teens reported that they carried the passport in their wallet or pocket wherever they went. Participant CH-007 noted: “[In my] Wallet, it was with me skiing today actually”. The participant who did not have the MyHealth Passport with him knew where it was and reported that he did not keep it with him because he did not have a wallet:

Nurse: Thats great! Where is your passport right now?

CH-058: i have it in my room in a safe area where it wwont get wrecked

Nurse: Do you ever carry a wallet with you?

CH-058: no not realyy though i probably shoul

Nurse: Well think about bc then you would have a place for your passport.

CH-058: ya good idea

Also of note was one teen who kept the MyHealth Passport in their pocket, but who subsequently reported putting the MyHealth Passport in his wallet:

Nurse: Where is your health passport right now?

Nurse: The one we did together last Monday. I remember putting it in your back pocket.

CH-061: In my back pocket lol

Nurse: That same back pocket? Or in the pocket of the pants you are now wearing?

CH-061: In the ones Im wearing right now

Nurse: Good work!! Has it made it to your wallet? Or maybe you dont carry your wallet with you all the time?

CH-061: Yeah I wear it all the time

#### Have You Used Your MyHealth Passport or Shown It to Anyone Else?

All teens reported that they had shown their MyHealth Passport to at least one person, and over half (7/13, 53.8%) of the teens had shown it to at least 2 people. One particular example was interesting because of what the teen considered to be a small number of people: CH-059: “Umm not to too many people maybe like 10-15 people”. Most teens showed their MyHealth Passport to family (69%) or friends (46%): CH-006: “I love showing it to people who mean a lot to me :)”. Only one teen indicated that they had used it in a health care setting. CH-063: “…only for doctor appointment when they ask what meds I take”. This was to be expected, as the text messaging occurred only 1 week after the MyHealth Passport had been created with the teen. One teen reported showing her MyHealth Passport to her coworkers. Using the MyHealth Passport in a work environment was a use of the MyHealth Passport not initially apparent to the researchers but one that could be useful for teens.

### Nature of the Texting Interaction

#### Formality of Language

Both formal and informal language was used by the nurses and teens, often within the same text message. Some teens opted to use full sentences that conveyed more formal language. For example, one teen, whose friend was with her during the clinic intervention with the nurse, reflected on her experience as follows: CH-006: “…We had a long talk on the way home it was a very positive opportunity for both of us”. Other teens opted to use shorter sentences and abbreviations of words. CH-017: “K thank you”. Most teens used a combination of formal and informal language throughout the interaction.

Our analysis of the texting interactions identified six categories of informal language: contractions, abbreviations, emoticons, text talk, multiple punctuation, and informal words. Both nurses and participants used contractions. Abbreviations were also common and included those that are in common general use (eg, OK) and more unique abbreviations such as birth control, which was shortened to “BC”. Text talk was the shortening of words and phrases frequently used in text messaging such as “LOL”, “NM”, or “u” for the word “you”. Text talk was mostly used by the participants as were emoticons. Teens also used informal words such as “Ya” and “Umm…” in their text messages. Multiple punctuation was exclusively used by the nurses, for example, “Excellent! Spring break, eh!?!”. Table 2 summarizes the informal language used by the nurses and participants.

**Table 2:** Instances of informal language in texting interactions.

Informal language	Nurse (n=72) n (%)	Participant (n=78) n (%)
Contraction	52 (72)	27 (35)
Abbreviation	14 (19)	19 (24)
Emoticons	0 (0)	10 (13)
Text talk	2 (3)	13 (17)
Multiple punctuations	4 (6)	0 (0)
Informal words	0 (0)	9 (11)

#### Correctness of Language

Spelling, grammatical, and typing errors were coded. Both nurses and participants appeared to express and understand ideas without difficulty despite the errors, but understanding of the texting interaction was not assessed. Spelling errors were deviations from the conventionally accepted spelling of a word, excluding errors caused by typographical errors (see below). Spelling errors occurred only two times. This was expected as most phones have spellcheck and autocorrect functions.

Grammatical errors were errors of syntax or morphology in the composition of clauses, phrases, and words. Such errors were common for both nurses and participants and are summarized in Table 3.

Typing errors occurred in 5 of the nurses’ messages and 11 of the teens’ messages. There were three categories of typing errors. The first was the omission of a letter unlikely due to a spelling error or text talk (eg, “shoul” for the word “should”) and accounted for 25% of typing errors. This is in contrast to when letters were dropped purposely to shorten words in text messages such as “till” for “until”. The second category was repetition of a letter within a word, such as “bbrought” or “wwont” and accounted for 37.5% of typing errors. The last category was the insertion of an improper word into a sentence and accounted for 37.5% of typing errors. This phenomenon sometimes occurred when a typing error was made and the phone’s autocorrect allowed the incorrect word as it was the correct spelling of another word, for example, “Can u plead get me the information for the Edmonton clinic”.

**Table 3:** Presence of grammatical errors by both researchers and teens.

Grammatical errors			Nurse (n=113) n (%)	Participant (n=345) n (%)	
**Errors of capitalization**					
	Omission		7 (6)		68 (20)
	Improper use		3 (2.5)		0 (0)
**Errors of punctuation**					
	**Omission**				
		Period	34 (30)		146 (42)
		Comma	35 (31)		99 (29)
		Apostrophe	0 (0)		13 (4)
		Dash	0 (0)		2 (1)
	**Improper use**				
		Period	1 (1)		0 (0)
		Comma	0 (0)		4 (1)
		Dash	13 (11)		0 (0)
**Missing words**					
	Verb		3 (2.5)		1 (0.3)
	Preposition		2 (2)		0 (0)
	Pronoun		3 (2.5)		1 (0.3)
	Subject		0 (0)		1 (0.3)
**Miscellaneous**					
	Verb-tense agreement		1 (1)		1 (0.3)
	Plural		2 (2)		0 (0)
	Starting sentence with a conjunction		4 (3.5)		3 (1)
	Homonym confusion		0 (0)		4 (1)
	Use of pronoun in place of adverb		1 (1)		1 (0.3)
	Use of improper preposition		2 (2)		0 (0)
	Use of pronoun in place of adjective		1 (1)		0 (0)
	Reverse word order		1 (1)		0 (0)
	Adjective used instead of adverb		0 (0)		1 (0.3)

#### No or Few Questions

Although invited, and even strongly encouraged, to contact the nurse with any questions or concerns regarding the clinic session, no teen contacted the nurse in the 7 days following the intervention. In all cases, it was the nurse who initiated contact with the teen. This was also true for email and phone interactions. Even when prompted with a scripted question, “Do you have any questions for me?”, only one teen responded with a question.

CH-061: Hey

CH-061: I have a question for you

Nurse: Let me hear it!

Nurse: And then Ill text you a question. You go first.

CH-061: Ok lol its just kinda but Im I able to drink energy drinks?

Nurse: Good question.

CH-061 also asked questions about pain he was experiencing.

Nurse: So anything else I can help you with. Any questions?

CH-61: Umm yeah I have one question..... sometimes I have a sharp pain it my lower stomach and I dont know what it is.

The nurse asked a few questions about the pain and then referred the teen to their general practitioner to follow up with any concerns.

The lack of questions received from teens led the nurse to ask if the participant would like more information on a topic related to their particular clinic intervention in 46% of conversations. For example, one teen had previously asked a nurse in the CHAPTER intervention if they were able to get a tattoo. The nurse used the texting interaction to expand on some of the risks associated with tattoos. Nurse: “I did find out a bit more about tattooing and the risk of endocarditis. Can I email that to you?” These nurse-initiated prompts led to more dialogue with the teen in all but one case. In this instance, the teen had to go to a basketball banquet and ended communication after the nurse asked if the teen wanted more information on cardiomyopathy. Communication with this teen was not re-established.

The theme of No or Few Questions was exemplified by one teen’s reported actions regarding his CHD. According to the 2007 American Heart Association guidelines regarding bacterial endocarditis prophylaxis [73], this teen no longer required antibiotics when going to the dentist. During the clinic intervention, the nurse realized that the teen was not sure about antibiotics so she had suggested that the teen ask his mom what the pediatric cardiologist had most recently recommended. The nurse followed up on this during the texting interaction:

Nurse: Did you have a chance to ask your mom about whether you are to have antibiotics?

Nurse: When you go to the dentist.

CH-058: yes i did she said i always have to take them cause off what i have but i didnt take any today

Nurse: You went to the dentist today?

CH-058: ya for a filling

CH-058: i had a dentist appointment today

Nurse: And who decided that you wouldnt have antibiotics thus time?

Nurse: This time?

CH-058: no idea i was just bbrought to the dentist i had no idea i was going until this morning

This teen’s apparent lack of initiative was not observed in the texting interactions with other teens in this study.

### Interaction With Health Care Providers

#### Overview

During the clinic intervention, nurses also provided information regarding how to communicate effectively with health care providers (HCPs). This was a point for follow-up during the texting interaction. Seven of the texting interactions (54%) concerned interaction with HCPs.

Analysis of the texting data indicated that one of the teens continued to have difficulty remembering the medical term for their CHD. During the texting interaction, the nurse encouraged her to use an abbreviation to more easily communicate with HCPs. Nurse: “Why don’t you just say T-G-A. That is the short form for Transposition of the Great Arteries. What do you think about that?” The nurse followed up with the teen on the telephone to have her practice pronouncing the name of her heart condition and encouraged the teen to use this terminology in her next clinic visit. Additionally, five of the interactions in some way focused on following up with the teen regarding their ability to connect with general practitioners and adult CHD cardiologists as needed. One teen was headed to summer camp, and the nurse highlighted the importance of having her family doctor’s phone number should problems arise.

Nurse: One more thing - did you find [primary physician’s name] phone # to put on your health passport?

CH-017: Oh i will

Nurse: Good! Do it while you remember.

CH-017: Yes

Nurse: Ok - that's all for now. Thanks!

Although no teens initiated a second texting interaction with the nurse for the purpose of asking questions, one teen texted the nurse when she returned to the hospital for a diagnostic test. She had completed questionnaires from the CHAPTER Study and wanted to return them directly to a study team member. The nurse contacted the research coordinator to retrieve the questionnaires from the teen and also made contact with the teen while she was waiting for her test to follow up on concerns that the teen had shared during the clinic intervention and texting.

#### Texting Teens in Transition

Because text messages can be responded to at any time, the teens transitioned through their after school and late afternoon activities while responding to the nurse-initiated text messages. To analyze temporal aspects of text messages, the unit of analysis was a conversation that began with the intervention nurse introducing themselves and ended with the nurse saying goodbye to the teen. There were 14 of these conversations. Regarding time of day, the earliest time for the texting interaction to begin was 2:56 pm and the latest start time for texting between the teen and the nurse was 7:40 pm The end time of the interaction ranged from 5:30 to 9:40 pm.

Both teens and nurses took advantage of their ability to respond over time. Conversations were an average of 67 minutes in length, and there was an average of 20 text messages an hour in each conversation. Teens were able to take part in many activities such as time with friends, sporting activities, attending events, and driving while fitting the text messaging in between these activities. For example, a nurse was able to schedule the interaction around a teen’s social activity. Nurse: “Can I give you a quick call to hear you say TGA?” CH-003: “Kk but can u call me at 9 bec i am at my friends right now”*.* The nurse was then able to call at 9 pm to continue the interaction.

Likewise, the nurses were engaged between work and home activities during the late afternoon and early evening texting interactions. Research field notes written by GR, the nurse who did most of the texting interactions indicated that she was involved in various activities when the texting occurred:

As I was texting back and forth with CH-016 I came up with a journal article title: “Texting teens in transition” – they are in transition in their life and they are also texting you as they move from place to place, activity to activity and I am texting them as I am moving through life – in the grocery store, walking home,…watching my niece run a race.

FN CH-016

#### Texting as a “Jumping-off Point”

In the data, there were occurrences of the nurses supplementing texting communication with the teens via email (15% of interactions), phone calls (8% of interactions), and websites (8% of interactions). For example, when a teen wanted to see what a baby with a heart condition looked like, the nurse guided the participant to a blog where a family chronicled their experience of having a child with the same heart condition as the participant.

CH-061: How are you?

*Nurse: Good. Wanted to check to see if you ever looked at that website about the baby with* [cyanotic congenital heart lesion] *- your heart problem.*

CH-061: Yeah I did thanks for that

The nurse then used this jumping-off point to ask the teens some questions to deepen the interaction:

Nurse: What did you think of it? Any surprises?

CH-061: Yeah I was surprised to see that I was just like that

Nurse: How did that make you feel seeing the little baby with all the tubes?

CH-061: I was really sad! :(

Nurse: Did you talk to anyone about this?

CH-061: No

Nurse: How are you feeling now?

CH-061: What do you mean? Like about the baby?

Nurse: About having had such a big operation when you were a baby?

CH-061: I feel like I was scary for my family and friends

Additionally, during the interaction with the teen who could not pronounce the name of her CHD, the nurse followed up with phone call to engage the teen in pronouncing the name of her heart condition. These examples illustrated that texting was not always appropriate but served as a reliable method to initiate other forms of communication. The nurse also emailed some teens in conjunction with the texting, including emailing information about selected topics, as relevant.

#### Affirmation

Affirmation of the teens was a recurrent theme in the texting data. One teen was affirmed for his insight after commenting on how the disease (during his infancy) had negatively affected his family CH-61: “I feel like I was scary for my family and friends” Nurse: “Yes- you would given them a big scare! That’s a good insight.” The nurse affirmed 2 (15%) teens for their apparent maturity, 2 (15%) for asking appropriate questions, 2 (15%) for demonstrating insight, and 8 (62%) for having the MyHealth Passport in their wallet. The following quote demonstrates a common affirmation given by the nurses. Nurse: “It was good to meet with you. I was impressed by your understanding of your [condition], meds, need for follow-up...Keep up the good work!”

#### Education

Due to the fact that all but one of the teens did not ask questions of the nurse without prompting, information was provided to 6 of the participants (46%) in a more educational fashion with information tailored to what had occurred during the in-person CHAPTER Study intervention. Topics suggested for further education included understanding the nature of their CHD, birth control, tattoos, heredity of a cardiac disease (8%), dental visits (8%), alcohol intake (8%), health care systems (8%), and use of energy drinks (8%). Even the inheritability of CHD, a potentially sensitive issue for the teens who generally did not realize that there was a chance that their offspring could be born with CHD, was addressed:

Nurse: One piece of information that you can put away for the future is about the chances of your kids having a heart problem.

Nurse: Its a little higher that I told you. I asked [pediatric cardiologist] about it.

CH-007: Okay sounds goof

Nurse: Do you want me to tell you more about this or do you want to talk with your doctor next time?

CH-007: Ya should be fun

Nurse: Ok well text back if you have questions

CH-007: You can tell me more sure thanks. So its a high chance they[offspring] will have it[CHD]?

Nurse: No not a high chance.In fact its a high chance they wont have it.

CH-007Okay thats what i thought, thats good!

Nurse: 85-90% that their hearts will be fine.

CH-007: Good to hear, thanks

Nurse: But with a 10-15% chance of having a heart problem you will want talk about this when you thinking about having kids. Ok?

CH-007: Ya ofcourse

**Figure 3:**
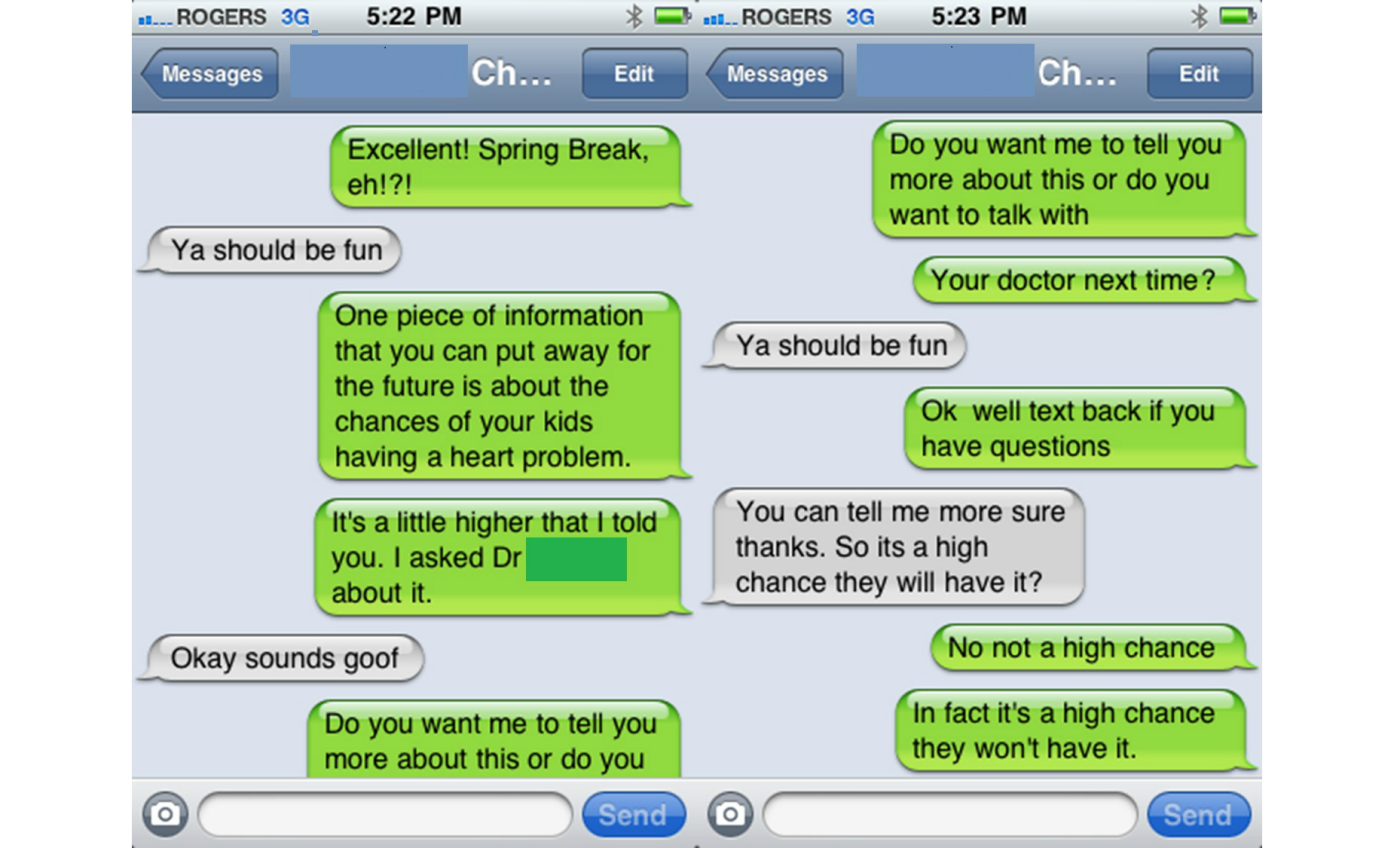
Out-of-sequence texts.

Given the back and forth nature of the texting interactions and the reality that teens were likely simultaneously texting others, the messages were sometimes out of sequence or did not seem to fit (eg, Ya should be fun posted twice; see Figure 3). In this situation, the nurse made sure that the teen was open to more information by offering the teen to “text back if you have questions”. This was also reflected in the nurse’s field note:

As I was texting CH 007 I was trying to imagine what kind of setting he was in. He had been skiing today – so is he in the car? Is he hanging out with friends? At home? Is this the time or is he in a place, physical and otherwise, to be talking about heart related things? He was upbeat in his responses to me and at one point I felt that he texted me the wrong response – “Ya should be fun” right after I was trying to figure out if he was open to hearing more about genetics and his CHD. I was willing to back off and then the next text showed his openness to hearing more: “You can tell me more sure thanks. So its a high chance they will have it?”

FN CH-007

This interaction and other similar interactions were mostly guided by the nurse instead of being guided by the teen as planned but with sensitivity to the teen’s openness to education that may be beneficial to the teen. Again, the nurse reflected on this in a field note:

I felt satisfied with how the texting interactions unfolded and I was able to put a positive spin on the numbers which I felt was appropriate given the nature of our interaction (texting during Spring Break) and his age and stage of life. I believe that our goal is to plant the seed for his future consideration.

FN CH-007

### Benefits and Drawbacks of Texting

#### Benefits and Risks

Through analysis of the interaction between youth and the nurse in texts, benefits and drawbacks were identified in terms of the use of texting as a communication modality.

Contact by texting was established among 81% of study participants. Both the teen and the nurse made use of texting and its associated ability to respond in the immediate or over time. Of note, many interactions took place over several hours with both parties responding at a time convenient to them. For example, many teens were able to work in the interactions between daily activities. CH-061: “Its not heart related[?]” Nurse: “Not likely. That’s why I asked about your bowels. But if you are concerned [patient’s primary cardiologist] would be the one to see.” CH-61: “Ok thanks but I got to go I’m about to go work out haha”. Another benefit is that the information was delivered in “byte-sized” amounts that were directly related to teen’s questions; the ability for the teen to guide discussion with their questions allowed for the discussion of a wide variety of topics.

#### Drawbacks

Drawbacks were also discovered upon analysis of the study transcripts. Because texting has inherent limits on the number of characters in each message, it may not always be a good medium for in-depth conversations; however, as seen above, in-depth interactions (although perhaps truncated comments) were possible. Accordingly, nurses often supplemented text messages with information delivered through other communication modalities. Another drawback identified was that participants may have been distracted during the interaction as they were able to continue other activities while texting, thus contributing to some of the shorter back and forth interactions. Nurse: “Did I ask you if anyone else in your family has a heart problem like your? Cardiomyopathy?” CH-63: “No sorry but I have to go my basketball banquet really soon so I need to change and drive there”. Nurse: “Ok - off you go!!” The last drawback that was found in the data is that the interaction could be perceived as invasive. The field note following this interaction indicated that the nurse felt bad that she had been texting with the teen as he was getting ready to go to a special event. Review of all of the texting data demonstrated that the nurse only sometimes asked the teen if it was a good time to text. On a positive note, as soon as the teen indicated that they had to move on to another activity, the nurse was receptive and terminated the interaction quickly as seen in the above example.

## Discussion

### Principal Findings

The use of text messaging has potential to contribute to communication between health care providers and teens and young adults. This study does not claim that texting is the best modality for follow-up, but rather seeks to begin the investigation of using texting in the pediatric cardiac population as a potential tool augmenting communication and patient monitoring. The majority of participants in this study could be contacted by text messaging and texting was the preferred mode of follow-up among teens. This seems to indicate that texting could be an appropriate modality of contact with this population and warrants further study. Texting also served well as a method of collecting data on how effective the clinic-based intervention was at promoting use of the MyHealth Passport. Data were collected easily at the convenience of the teen and nurse, and because this data was already in electronic form, they were easily captured for analysis.

Despite the ubiquitous nature of texting among adolescents, all of the texting interactions in this study were initiated by the nurse even when the teens were advised that they were welcome to text the nurse with any questions. Additionally, during the nurse-initiated texting interaction, the teens had no or few questions and required further interaction with the nurse to deepen the conversation. This could be indicative of the interaction being nurse driven rather than teen driven. Given the considerable risk of teens with CHD being lost to follow-up [77,78], however, and the grave implications this has for their future health outcomes [79], this convenient and preferred method of interaction warrants further investigation in the context of intervention research and evaluation of transition programs in pediatric cardiology.

In regards to the nature of the texting interaction, this study adds to the literature. Although the original plan of the study was to interact completely through text message, it was found that some of the interactions resulted in the use of email, blogs, telephone, and other modes of communication. This finding is useful because it suggests that texting may serve as an initial mode of contact and as a way to connect teens to other resources, what we have called a “jumping-off point”. This being said, the information shared between the nurse and the teen within text messages was varied and important. Topics discussed included reviewing the name of the teen’s heart condition, birth control, tattoos, heredity of heart conditions, dentist visits, alcohol and energy drinks, and seeing a baby with the same heart condition. As various topics were addressed during a texting interaction, we were assured that texting was an appropriate medium for such specific education.

### Limitations

Although this study provides useful information, it does have limitations. First, this study includes non-uniform interactions with a limited sample of 13 participants. For this reason, the findings are not representative of teens with CHD in general but offer information regarding the usefulness and viability of this type of texting interaction.

Second, this study required that teens had access to a cell phone. This was not a problem for the participants in this study but could be an issue for those working with disadvantaged populations. The same is also true for populations that do not consider cell phones appropriate for teenagers for social or cultural reasons. Researchers working with these populations may consider providing low-cost cell phones to their participants for health-related communications only, as has been done in other studies [7,14,15].

There is some question of whether or not these types of interactions, with a full back-and-forth interaction between nurse and teen, could benefit from more established protocols as two knowledge syntheses suggest [80,81]. Findings from this qualitative analysis provide direction for a protocol about the content of and approach to texting teens following a clinic-based intervention to facilitate their readiness for transition from pediatric to adult care. This guideline will provide nurses with direction for interacting with teens who may not respond well to open interaction, such as the passiveness demonstrated by the teens in this study. Implementing and evaluating established protocols for texting may allow studies to draw more powerful conclusions. Identifying a solid theoretical framework to inform the texting interactions is needed and supported by a related meta-analysis [80].

While this study does appear to indicate that texting can result in more in-depth interactions than have previously been reported in studies involving text messaging [1-6,8-13,16-54], this type of intervention is unlikely to be feasible in its current state at a large-scale organizational level. The interactions in this study were open-ended, featuring full discussions between the teen and the nurse that occurred in the late afternoon and early evening, differing from many of the studies that have occurred in the area that are mostly automated. There is some doubt as to whether this open-ended type of texting interaction is feasible because of the time and staff necessary to provide this service to all patients transitioning from pediatric to adult cardiology. There are several ways to lessen the demands of implementing such an intervention. Those wishing to implement a program using full back-and-forth interactions may consider automating some of the interaction. The research of Reback et al [14,15] included both pre-written automated messages and real-time personalized text messages to study staff. In this study, automated texting was used both to establish contact with participants through an introductory message and to also deliver pre-written educational messages. This type of strategy would reduce time and labor constraints and would also allow for more in-depth interaction than automated texting alone. Additionally, we recommend that the nurse determine the teen’s openness to text during the day, for example, during the lunch break at school or another part of the school day when the teen can direct some attention to the text messages. More research with larger sample sizes is needed to explore the feasibility of text messaging in clinical settings.

This study identified possible risks of using text messaging in this manner. In addition to the benefits and drawbacks discussed, there is the issue of confidentiality. Although this study encountered no known breach of confidentiality, there is an inherent risk in communicating through non-secure channels. The authors of this study would recommend that there be some teaching about security and mobile devices before communicating with teens through text messaging. Teens would benefit from using pass codes on their mobile devices and deleting messages after the interaction has been completed. Content management systems to which texting data are immediately transferred are being developed [82]. Another risk is that teens feel that they do not have a choice about engaging in the texting interaction given the immediacy of the modality. The CHAPTER Study protocol now includes a question by the nurse at the beginning of the texting interaction (“is this a good time to ‘talk’?”) with a special point made to ensure that the teen is not driving while texting.

One final ethical concern is the possibility that a serious issue or concern could be raised in a text and the youth is not available for, or engaged in, follow-up or is not reachable perhaps due to their being mobile and/or engaging in texting only in a brief moment or engaging in truncated conversation. This did not prove to be a problematic issue in this study; however, the brevity of texting and the mobility in which it is often used, potentially renders subsequent follow-up difficult. But as a tool for maintaining communication with youth with chronic illness, texting appears to add augmentative value to clinical follow-up, particularly for populations that value this flexible modality of communication.

### Conclusion

Text messaging offers an important augmentative method of communicating with patients that could assist in care monitoring and follow-up. In this study, text messaging was useful for collecting data about the effectiveness of the clinic-based intervention with teens with CHD. Text messaging also resulted in conversations with the teens that in some cases were reasonably in-depth and meaningful to their health and well-being, especially when combined with other forms of communication. The use of text messaging, in a manner that results in full conversations with the patient, requires more study and could benefit from protocols and a solid theoretical foundation that would ground and potentially standardize the interaction so that firmer conclusions of this novel intervention could be drawn. Toward that end, this study offers evidence of benefit and viability.

## Multimedia Appendix 1

Poster presentation 2012.

## Abbreviations

CHD: congenital heart disease
CHAPTER: Congenital Heart Adolescents Participating in Transition Evaluation Research
FN: field notes
HCPs: health care providers
