# Correction: Texting Teens in Transition: The Use of Text Messages in Clinical Intervention Research

**DOI:** 10.2196/mhealth.4250

**Published:** 2015-02-02

**Authors:** Gwen R Rempel, Ross T Ballantyne, Joyce Magill-Evans, David B Nicholas, Andrew S Mackie

**Affiliations:** ^1^Athabasca UnversityFaculty of Health DisciplinesAthabasca, ABCanada; ^2^University of AlbertaFaculty of NursingEdmonton, ABCanada; ^3^University of AlbertaDepartment of Occupational TherapyEdmonton, ABCanada; ^4^University of CalgaryFaculty of Social WorkCalgary, ABCanada; ^5^University of AlbertaFaculty of Medicine and DentistryEdmonton, ABCanada

The authors of “Texting Teens in Transition: The Use of Text Messages in Clinical Intervention Research" (JMIR *mHealth uHealth* 2014; 2(4):e45) neglected on submission to acknowledge the support and assistance of Dr Miriam Kaufman from the Good 2 Go Transition Program, The Hospital for Sick Children, in the use of the MyHealth Passport. The authors also did not update reference 72 (which they previously cited as Web reference discussing MyHealth Passport, http://www.webcitation.org/6GIIRwHSd) to the following article: Wolfstadt J, Kaufman A, Levitin J, Kaufman M. The use and usefulness of my health passport: an online tool for the creation of a portable health summary*. Int J of Child and Adolesc Health*. 2011;3(4):499-506. These errors have been corrected in the online version of the paper on the JMIR MHealth and UHealth website on February 2nd, 2015, together with publishing this correction notice. A correction notice has been sent to PubMed and the correct full-text has been resubmitted to Pubmed Central and other full-text repositories.

