# Supplementary material for: Texting Teens in Transition: The Use of Text Messages in Clinical Intervention Research
Source: JMIR Mhealth Uhealth. 2014 Nov 6;2(4):e45. doi: 10.2196/mhealth.3232 (PMC4260009; doi:10.2196/mhealth.3232)
Supplement: Supplementary file 1 [file mhealth_v2i4e45_app1.pptx]

## Slide 1
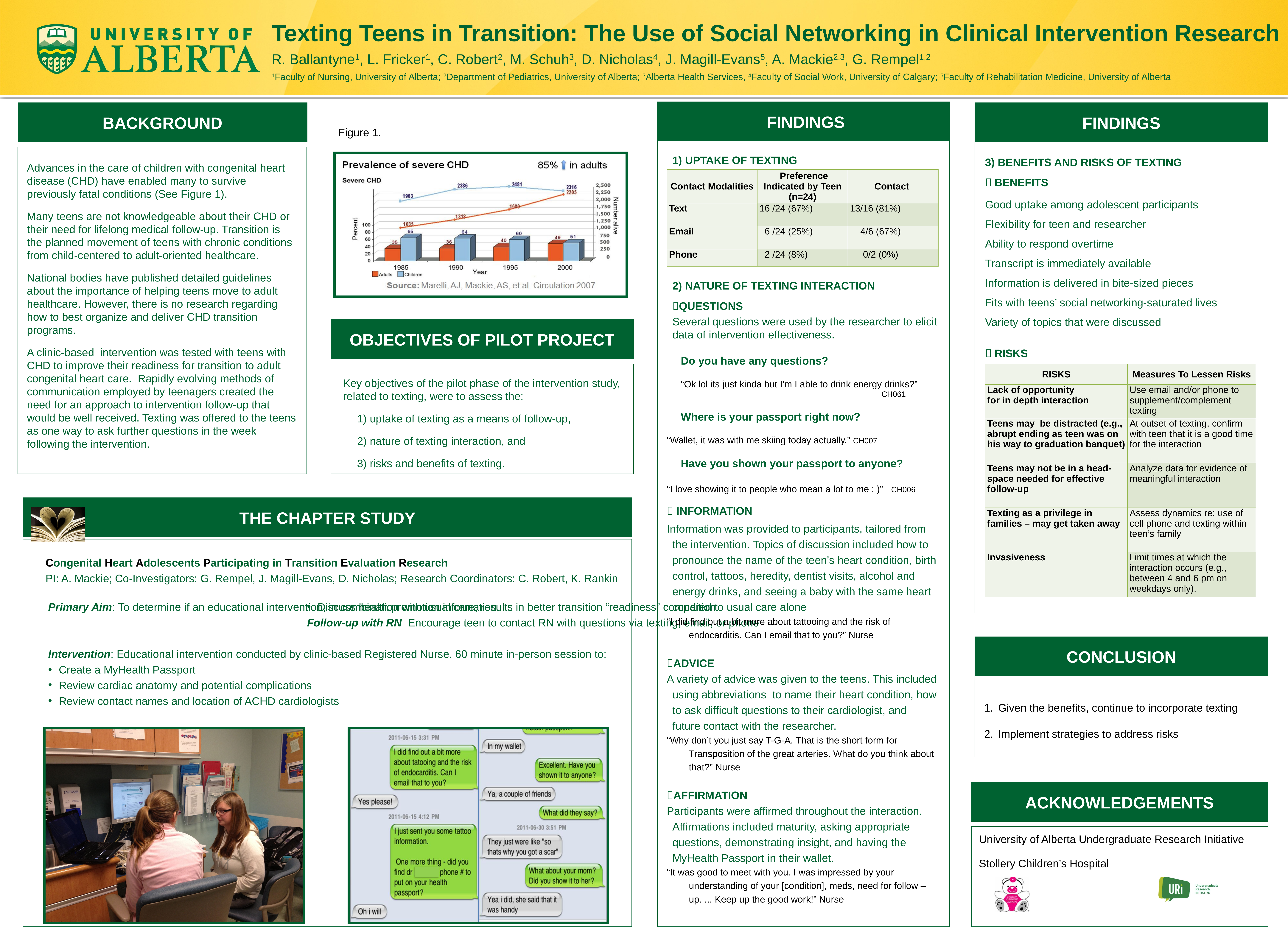

Texting Teens in Transition: The Use of Social Networking in Clinical Intervention Research
R. Ballantyne1, L. Fricker1, C. Robert2, M. Schuh3, D. Nicholas4, J. Magill-Evans5, A. Mackie2,3, G. Rempel1,2
1Faculty of Nursing, University of Alberta; 2Department of Pediatrics, University of Alberta; 3Alberta Health Services, 4Faculty of Social Work, University of Calgary; 5Faculty of Rehabilitation Medicine, University of Alberta
 FINDINGS
BACKGROUND
Figure 1.
FINDINGS
1) UPTAKE OF TEXTING
2) NATURE OF TEXTING INTERACTION
QUESTIONS
Several questions were used by the researcher to elicit data of intervention effectiveness.
Do you have any questions?
“Ok lol its just kinda but I'm I able to drink energy drinks?” CH061
Where is your passport right now?
“Wallet, it was with me skiing today actually.” CH007
Have you shown your passport to anyone?
“I love showing it to people who mean a lot to me : )” CH006
 INFORMATION
Information was provided to participants, tailored from the intervention. Topics of discussion included how to pronounce the name of the teen’s heart condition, birth control, tattoos, heredity, dentist visits, alcohol and energy drinks, and seeing a baby with the same heart condition.
“I did find out a bit more about tattooing and the risk of endocarditis. Can I email that to you?” Nurse
ADVICE
A variety of advice was given to the teens. This included using abbreviations to name their heart condition, how to ask difficult questions to their cardiologist, and future contact with the researcher.
“Why don’t you just say T-G-A. That is the short form for Transposition of the great arteries. What do you think about that?” Nurse
AFFIRMATION
Participants were affirmed throughout the interaction. Affirmations included maturity, asking appropriate questions, demonstrating insight, and having the MyHealth Passport in their wallet.
“It was good to meet with you. I was impressed by your understanding of your [condition], meds, need for follow – up. ... Keep up the good work!” Nurse
3) BENEFITS AND RISKS OF TEXTING
 BENEFITS
Good uptake among adolescent participants
Flexibility for teen and researcher
Ability to respond overtime
Transcript is immediately available
Information is delivered in bite-sized pieces
Fits with teens’ social networking-saturated lives
Variety of topics that were discussed
 RISKS
Advances in the care of children with congenital heart disease (CHD) have enabled many to survive previously fatal conditions (See Figure 1).
Many teens are not knowledgeable about their CHD or their need for lifelong medical follow-up. Transition is the planned movement of teens with chronic conditions from child-centered to adult-oriented healthcare.
National bodies have published detailed guidelines about the importance of helping teens move to adult healthcare. However, there is no research regarding how to best organize and deliver CHD transition programs.
A clinic-based intervention was tested with teens with CHD to improve their readiness for transition to adult congenital heart care. Rapidly evolving methods of communication employed by teenagers created the need for an approach to intervention follow-up that would be well received. Texting was offered to the teens as one way to ask further questions in the week following the intervention.
| Contact Modalities | Preference Indicated by Teen (n=24) | Contact |
| --- | --- | --- |
| Text | 16 /24 (67%) | 13/16 (81%) |
| Email | 6 /24 (25%) | 4/6 (67%) |
| Phone | 2 /24 (8%) | 0/2 (0%) |
OBJECTIVES OF PILOT PROJECT
Key objectives of the pilot phase of the intervention study, related to texting, were to assess the:
1) uptake of texting as a means of follow-up,
2) nature of texting interaction, and
3) risks and benefits of texting.
| RISKS | Measures To Lessen Risks |
| --- | --- |
| Lack of opportunity for in depth interaction | Use email and/or phone to supplement/complement texting |
| Teens may be distracted (e.g., abrupt ending as teen was on his way to graduation banquet) | At outset of texting, confirm with teen that it is a good time for the interaction |
| Teens may not be in a head-space needed for effective follow-up | Analyze data for evidence of meaningful interaction |
| Texting as a privilege in families – may get taken away | Assess dynamics re: use of cell phone and texting within teen’s family |
| Invasiveness | Limit times at which the interaction occurs (e.g., between 4 and 6 pm on weekdays only). |
THE CHAPTER STUDY
Congenital Heart Adolescents Participating in Transition Evaluation Research
PI: A. Mackie; Co-Investigators: G. Rempel, J. Magill-Evans, D. Nicholas; Research Coordinators: C. Robert, K. Rankin
Primary Aim: To determine if an educational intervention, in combination with usual care, results in better transition “readiness” compared to usual care alone
Intervention: Educational intervention conducted by clinic-based Registered Nurse. 60 minute in-person session to:
Create a MyHealth Passport
Review cardiac anatomy and potential complications
Review contact names and location of ACHD cardiologists
Discuss health promotion information
Follow-up with RN Encourage teen to contact RN with questions via texting, email, or phone
CONCLUSION
Given the benefits, continue to incorporate texting
Implement strategies to address risks
ACKNOWLEDGEMENTS
University of Alberta Undergraduate Research Initiative
Stollery Children’s Hospital
